# Clinical utility of lung ultrasound in differentiating and monitoring complicated pediatric pneumonia: a prospective diagnostic accuracy study

**DOI:** 10.1186/s13052-026-02341-3

**Published:** 2026-10-03

**Authors:** Rehab Elmeazawy, Ebtsam Ahmed Attia, Osama Toema, Nancy Mohamed Elgharabawy, Ahmed Harkan, Ahmed M. Elniny

**Affiliations:** Department of Pediatrics, Faculty of Medicine, Tanta University, Tanta, Egypt

**Keywords:** Lung ultrasound, LUS score, Pediatric pneumonia, Necrotizing pneumonia, Parapneumonic effusion, Diagnostic accuracy

## Abstract

**Background:**

Lung ultrasound (LUS) is an established diagnostic tool for pediatric pneumonia, yet its utility as radiation-free alternative to computed tomography (CT) for monitoring disease progression and differentiating complicated phenotypes, specifically necrotizing pneumonia (NP) and parapneumonic effusion (PPE), has not been prospectively validated in a single comparative cohort. This study aimed to evaluate the diagnostic and longitudinal performance of LUS, including a quantitative LUS score, in children with community-acquired pneumonia (CAP), CT-confirmed NP, and PPE, and to evaluate the diagnostic accuracy and longitudinal performance of LUS for identifying and monitoring complicated pediatric pneumonia, including its potential as a radiation-free follow-up tool.

**Methods:**

This prospective single-center diagnostic accuracy study enrolled 257 children diagnosed with CAP (*n* = 116), NP (*n* = 54), and PPE (*n* = 87). NP was confirmed by CT in all 54 cases; LUS was performed by trained operators at admission and on day five as a paired assessment against CT and chest radiograph findings. LUS qualitative features and quantitative scores were compared between groups and over time. Multivariable regression and ROC analysis were performed to assess diagnostic accuracy and identify predictors of clinical outcome.

**Results:**

At admission, NP and PPE showed significantly higher LUS scores and more frequent absent lung sliding and heterogeneous hypoechoic consolidation than CAP (*p* < 0.001). A LUS score cutoff of > 8 demonstrated strong diagnostic performance at both time points (admission AUC: 0.881, sensitivity 79.02%; Day 5 AUC: 0.861, specificity 93.86%), supporting its potential role as an early screening marker and as a radiation-free bedside monitoring tool during follow-up. Longitudinal LUS assessment demonstrated significant re-aeration by Day 5 (*p* < 0.001), evidenced by an increased proportion of separated B-lines (35.0% to 54.9%) and a reduction in lung consolidation. On multivariable analysis, higher LUS scores independently predicted longer hospitalization on both admission (β = 0.221, *p* < 0.001) and Day 5 (β = 0.215, *p* < 0.001), and elevated D-dimer on admission (β = 0.334, *p* < 0.001) and Day 5 (β = 0.386, *p* < 0.001).

**Conclusion:**

A LUS score > 8 showed promising diagnostic performance for identifying complicated pediatric pneumonia at admission and during Day 5 follow-up. Although internal bootstrap validation supported the stability of its performance, prospective validation in independent cohorts is required before implementation as a CT-sparing follow-up strategy.

**Supplementary Information:**

The online version contains supplementary material available at https://doi.org/10.1186/s13052-026-02341-3.

## Background

Pneumonia remains a major cause of morbidity and mortality among children worldwide. Although most cases of community-acquired pneumonia (CAP) have an uncomplicated course, a subset progresses to complications such as necrotizing pneumonia (NP), parapneumonic effusion (PPE), empyema, and lung abscess, which are associated with prolonged hospitalization and increased need for interventions. Early identification of these phenotypes is essential for timely, risk-based management [1–3].

Imaging plays a central role in evaluating pediatric pneumonia. Chest radiography (CXR), although widely used, has limitations in detecting and characterizing parenchymal and pleural complications [4, 5]. Computed tomography (CT) remains the reference standard for detecting necrosis and cavitation; however, concerns regarding radiation exposure and the need for sedation limit its repeated use, creating a need for safe bedside monitoring strategies [6].

Lung ultrasound (LUS) has emerged as a promising bedside modality with growing application across pediatric respiratory disease. Its advantages, real-time imaging, portability, repeatability, absence of ionizing radiation, and cost-effectiveness, make it particularly suited to serial monitoring in acutely ill children [7]. Specific sonographic patterns, including subpleural consolidation with dynamic air bronchograms, pleural line irregularities, and confluent B-lines, enable differentiation of bacterial from viral pneumonia and detection of complications such as pleural effusion and empyema [8, 9]. Notably, LUS may equal or surpass CT in characterizing pleural septations and guiding drainage procedures, positioning it as a high-value tool in the management of complicated cases [9].

Despite increasing evidence supporting LUS in pediatric pneumonia, prospective studies directly comparing quantitative LUS performance among CAP, CT-confirmed NP, and PPE with longitudinal follow-up remain limited. Previous studies have mainly assessed LUS at a single time point or included heterogeneous pneumonia populations, leaving its role in monitoring treatment response and differentiating complicated phenotypes insufficiently defined [10]. Critically, no prospective study has evaluated whether a validated LUS score can serve as a radiation-free monitoring tool for pediatric necrotizing pneumonia. Therefore, this study evaluates the utility of LUS in differentiating and longitudinally monitoring pediatric CAP, NP, and PPE, determining if quantitative LUS scores offer a reliable, CT-sparing follow-up strategy.

## Methods

This prospective study was conducted at the Pediatric Pulmonology Unit, Tanta University Hospital, Tanta, Egypt, a tertiary referral academic center providing specialized respiratory care for children and receiving referrals of complicated pneumonia from surrounding hospitals, between January 2023 and August 2025.

Consecutive eligible children admitted with a diagnosis of CAP and its complications were prospectively enrolled, with LUS performed at standardized time points alongside conventional imaging. The study was conducted in accordance with the Declaration of Helsinki, and ethical approval was granted by the Research Ethics Committee of the Faculty of Medicine, Tanta University (approval number 36264PR855/9/24). Written informed consent was obtained from the legal guardians of all participants prior to enrollment.

Children aged two months to 18 years admitted with CAP were eligible for inclusion. CAP was defined as the presence of clinical signs and symptoms of pneumonia in a previously healthy child who acquired the infection in the community, accompanied by radiological evidence of lung consolidation [11]. Children were prospectively assigned to one of three groups based on clinical, radiological, and, where applicable, CT findings established at or within 48 h of admission: Group 1, uncomplicated CAP (*n* = 116); Group 2, necrotizing pneumonia (NP; *n* = 54); and Group 3, parapneumonic effusion (PPE; *n* = 87).

PPE was diagnosed on the basis of clinical examination and chest radiograph or ultrasound findings of free or loculated pleural fluid in the context of pneumonia. NP was diagnosed in children presenting with progressive pneumonia refractory to appropriate antibiotic therapy, with CT chest demonstrating multiple necrotic foci within the consolidated lung parenchyma, CT confirmation was obtained in all 54 NP cases [12]. CT was performed according to predefined clinical criteria, including persistent or worsening fever despite adequate antimicrobial therapy, suspected pulmonary complications, or the need for further anatomical delineation and interventional planning. CT confirmation was obtained in all 54 children diagnosed with NP. The CT-confirmed NP criterion was pre-specified in the study protocol to ensure diagnostic consistency and to establish CT as the reference standard against which LUS follow-up performance was evaluated.

The study groups were mutually exclusive. Children with CT-confirmed NP were classified in the NP group regardless of the presence of concomitant parapneumonic effusion. The PPE group included only children with parapneumonic effusion without evidence of NP.

Children with chronic lung disease, immunodeficiency, and other comorbidities were excluded, and findings may not apply to these high-risk populations.

No formal priori sample size calculation was performed. Instead, the sample size was determined by the consecutive enrollment of all eligible children who met the inclusion criteria during the predefined study period.

### Clinical data collection

Demographic, clinical, laboratory, and imaging data were collected prospectively at admission and on day five. Variables included age, sex, weight, weight-for-age z-score, respiratory rate, presenting symptoms (including dyspnea which was defined as parent- or clinician-reported shortness of breath or difficulty breathing, recorded at presentation. Objective signs of increased work of breathing (tachypnea, retractions, nasal flaring, grunting, accessory muscle use, and cyanosis) were recorded as separate variables), affected lung laterality, and length of hospital stay.

Laboratory investigations included complete blood count (hemoglobin, total leukocyte count, absolute neutrophil count [ANC], absolute lymphocyte count [ALC], and platelets), and C-reactive protein (CRP). D-dimer was measured when clinically indicated in children with severe or complicated pneumonia and was analyzed as an exploratory parameter. Conventional imaging included posteroanterior and lateral chest radiographs at matched time points. Chest CT was performed using a pediatric protocol, with acquisition parameters adjusted according to patient age and body size to keep radiation exposure as low as reasonably achievable (ALARA). CT was obtained during hospitalization in children who met predefined clinical and/or imaging criteria suggestive of necrotizing pneumonia or in complicated cases requiring further anatomical evaluation or interventional planning. Intravenous contrast was administered only when clinically indicated at the discretion of the treating physician. CT findings served as the reference standard for the diagnosis of necrotizing pneumonia and for evaluating the diagnostic performance of LUS. Chest CT was not routinely repeated at Day 5 because of ethical concerns regarding radiation exposure in children. Therefore, no contemporaneous imaging reference standard was available for the Day 5 assessment.

### Lung ultrasound examination

LUS was performed at admission (Day 1) and on Day 5 using a portable ultrasound machine (Mindray DP-20, Mindray Bio-Medical Electronics Co., Ltd., Shenzhen, China) equipped with a high-frequency linear probe (7–12 MHz) and, where required for deeper structures, a low-frequency convex probe (3–5 MHz). All examinations were conducted at the bedside. The LUS operators were aware of the patients’ clinical presentation because examinations were performed as part of routine clinical assessment; however, they were blinded to chest CT and chest radiograph findings at the time of LUS acquisition and interpretation. Laboratory results and final diagnostic classification were not used in LUS interpretation. Each hemithorax was systematically scanned in six lung zones (anterior, lateral, and posterior; upper and lower), yielding a total of 12 lung zones, according to a standardized pediatric LUS protocol [13, 14]. Predefined sonographic features recorded at each examination included A-lines, B-lines, subpleural consolidation, consolidation with air bronchograms, tissue-like pattern, pleural effusion and its character, decreased lung sliding, heterogeneous hypoechoic areas, and hypoechoic lesions.

A quantitative LUS score was calculated for each patient at both time points by summing aeration scores across the 12 lung zones, as previously described [15]. This aeration-based scoring framework, originally described in adult critical-care populations, has since been adapted and validated in neonates, including for evaluating oxygenation and surfactant need in neonates on CPAP [16] and guiding surfactant therapy in late-preterm and older neonates [17], supporting its use as a semi-quantitative aeration measure in pediatric populations.

Each zone was assigned an aeration score from 0 to 3: **0 =** normal aeration with A-lines or fewer than three isolated B-lines; **1 =** multiple well-spaced B-lines; **2 =** confluent B-lines indicating severe interstitial involvement; and **3 =** lung consolidation with or without air bronchograms. The total LUS score therefore ranged from 0 to 36, with higher scores indicating greater loss of lung aeration. The number of involved lung segments was recorded separately.

Pleural effusions were classified according to their sonographic appearance using a previously published grading system [18]. Grade 1 represented a simple anechoic effusion without septations or loculations. Grade 2 represented a complex non-septated effusion characterized by echogenic fluid without septations. Grade 3 represented a complex septated effusion with multiple septations/loculations. Grade 4 represented a highly complex effusion with multiple septations and solid-appearing components comprising more than one-third of the collection. For analysis, Grades 3 and 4 were grouped as complex septated effusions.

All examinations were conducted by two trained pediatric pulmonologists, each with more than three years of dedicated LUS experience and a personal case volume exceeding 500 examinations. To assess interobserver agreement, both operators independently performed and interpreted LUS in a randomly selected 20% subset of participants (*n* = 52) at both Day 1 and Day 5, blinded to each other’s findings. Agreement was quantified using Cohen’s kappa (κ), interpreted as: <0.20 poor, 0.21–0.40 fair, 0.41–0.60 moderate, 0.61–0.80 substantial, and 0.81–1.00 almost perfect.

All LUS examinations were technically adequate for interpretation. No indeterminate LUS examinations were encountered; therefore, no examinations required repetition, exclusion, or alternative classification.

No indeterminate LUS examinations or reference standard results were encountered during the study. All enrolled participants had complete index test and reference standard data at both assessment time points; therefore, no data were missing and required imputation or exclusion from the analyses. No adverse events related to the LUS examination were observed during the study. Low-dose chest CT was performed only when clinically indicated, and no CT-related adverse events were observed.

### Statistical analysis

Data were analyzed using SPSS version 27 and MedCalc. Categorical variables were presented as counts and percentages and compared using the chi-square test or chi-square test for trend. Continuous variables were expressed as mean ± SD or median (IQR) depending on distribution, with normality assessed by the Shapiro-Wilk test. Comparisons between two groups used t-test or Mann-Whitney U test. To compare quantitative data between more than two groups, Kruskal Wallis test (for not normally distributed data) and one way ANOVA test (for normally distributed data) were used, with post hoc pairwise comparisons via Tukey HSD when applicable. Pearson or Spearman correlation coefficients were used to assess relationships between variables. Receiver operating characteristic (ROC) curve analysis was performed to evaluate the diagnostic performance of the LUS score and to determine the optimal cutoff using the Youden index. To assess the internal validity of the selected cutoff and reduce the risk of overfitting, internal bootstrap validation was performed using 1,000 bias-corrected and accelerated (BCa) bootstrap resamples. Bootstrap confidence intervals were calculated for the area under the ROC curve (AUC), the Youden index, and the optimal cutoff criterion. Linear regression was used to identify independent predictors. To account for multiple comparisons across the panel of LUS qualitative and quantitative features, statistical significance was additionally assessed using the Benjamini-Hochberg procedure to control the false discovery rate at Q = 0.05, applied separately within each table’s family of comparisons. Statistical significance was set at *p* < 0.05, with *p* ≤ 0.001 considered highly significant.

**Table 1:** Demographic and clinical characteristics of the studied groups

Lung ultrasound parameter	First day	Fifth day	*p* value	BH-adjusted q value
Lung ultrasound (LUS) score, median (IQR)	8 (5–13)	6 (3–12)	< 0.001*	< 0.001*
Number of involved lung segments, median (IQR)	5 (4–6)	4 (3–6)	< 0.001*	< 0.001*

*Statistically significant at raw *p* < 0.05 and remained significant after Benjamini-Hochberg false discovery rate correction (Q = 0.05). IQR: interquartile range

**Table 2:** Laboratory and inflammatory parameters among the studied groups

Lung ultrasound parameter	CAP (*n* = 116)	NP (*n* = 54)	PPE (*n* = 87)	*P* value	BH-adjusted q value
**First day findings,** ***n*** **(%)**					
Number of involved segments, median (IQR)	4 (4–6)	6 (5–6)	6 (5–6)	< 0.001*	< 0.001*
LUS score, median (IQR)	5.5 (5–7)	11.5 (7–14)	12 (11–14)	< 0.001*	< 0.001*
**Fifth day findings**,** n (%)**					
Number of involved segments, median (IQR)	3 (2–4)	6 (4.75–6)	5 (4–6)	< 0.001*	< 0.001*
LUS score (5th day), median (IQR)	4 (3–6)	12 (11–14)	12 (10–13)	< 0.001*	<0.001*

*Statistically significant at raw *p* < 0.05 and remained significant after Benjamini-Hochberg false discovery rate correction (Q = 0.05). IQR: interquartile range

## Results

### Baseline characteristics and clinical presentation

**Fig. 1:**
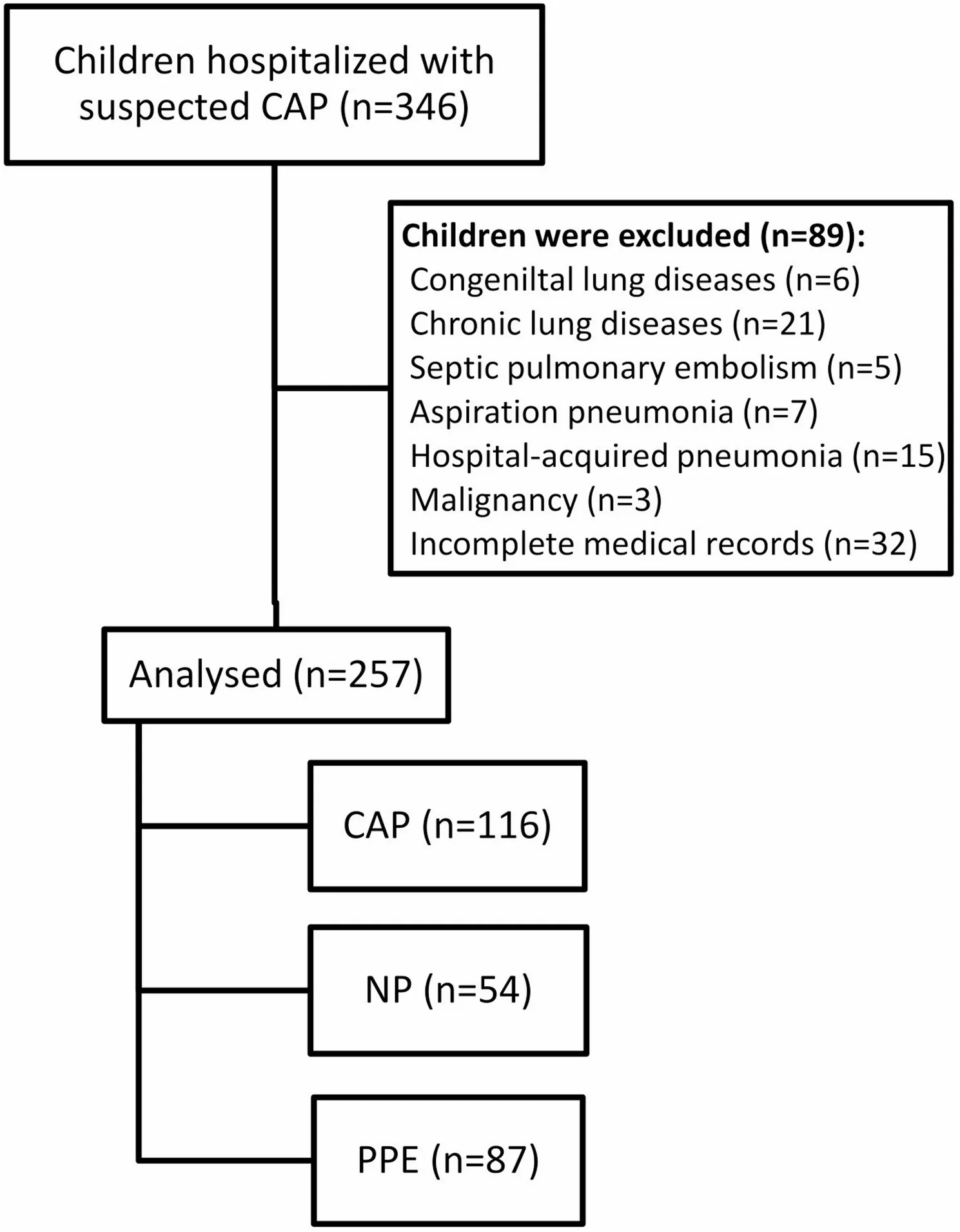
Flow chart of the studied patients

A total of 257 children were prospectively enrolled: 116 with CAP, 54 with CT-confirmed NP, and 87 with PPE (Fig. 1). The cohort was 57.2% female, with no significant sex difference across groups (*p* = 0.569). The three groups differed significantly in age (*p* = 0.003), with NP patients being the youngest (median 3 years, IQR 1.98–4.5), consistent with the known predilection of necrotizing disease for younger children. Weight and weight-for-age z-score were similarly lowest in the NP group (*p* = 0.007), suggesting greater nutritional vulnerability in this phenotype.

Fever was nearly universal (97.7%) and did not differ across groups. Among distinguishing clinical features, grunting was most prevalent in NP (53.7%, *p* < 0.001) and respiratory rate was highest in the NP group (52.74 ± 9.44 breaths/min, *p* < 0.001), together reflecting the greater physiological burden of necrotizing disease at presentation. Dyspnea was paradoxically more frequent in the CAP group (47.4% vs. 27.8% in NP and 31.0% in PPE, *p* = 0.014). Right lung involvement predominated across the cohort (63.8%), with bilateral involvement more common in CAP (*p* = 0.015).

Disease severity was reflected in length of hospital stay, which differed significantly across groups (*p* < 0.001). NP carried the greatest hospitalization burden, with a median stay of 16.5 days (IQR 11–21.25), compared with 13 days (IQR 8–15) for PPE and 7 days (IQR 5–9.75) for CAP, a gradient that paralleled both LUS scores and inflammatory markers, as described below (Table 1 ).

**Table 3:** Comparison of lung ultrasound scores and involved segment between the first and fifth days of admission

Variable	Total (*n* = 257)	CAP (*n* = 116)	NP (*n* = 54)	PPE (*n* = 87)	*P* value
**Sex, ** ***n*** ** (%)**					
• Female	147 (57.2)	66 (56.9)	28 (51.9)	53 (60.9)	0.569
• Male	110 (42.8)	50 (43.1)	26 (48.1)	34 (39.1)	
**Age (years)**, median (IQR)	4 (2.8–7)	4 (3–8)	3 (1.98–4.5)	4 (2.9–7)	0.003*
**Weight (kg)**, median (IQR)	15 (13–26)	16 (11–27)	14 (11–16)	16 (14-26.7)	0.007*
**Weight z-score**, median (IQR)	−0.39 (− 0.63, 0.62)	−0.34 (− 0.63, 0.79)	−0.51 (− 0.82 − 0.34)	−0.34 (− 0.53,0.71)	0.007*
**RR (breaths/min)**, mean ± SD	48.26 ± 10.28	46.08 ± 10.0	52.74 ± 9.44	48.38 ± 10.32	< 0.001*
**Clinical manifestations**,** n (%)**					
Fever	251 (97.7)	113 (97.4)	53 (98.1)	85 (97.7)	0.957
Cough	136 (52.9)	65 (56.0)	27 (50.0)	44 (50.6)	0.661
Dyspnea	97 (37.7)	55 (47.4)	15 (27.8)	27 (31.0)	0.014*
Accessory muscle use	94 (36.6)	46 (39.7)	14 (25.9)	34 (39.1)	0.187
Grunting	89 (34.6)	27 (23.3)	29 (53.7)	33 (37.9)	< 0.001*
Chest pain	34 (13.2)	16 (13.8)	4 (7.4)	14 (16.1)	0.325
Abdominal pain	64 (24.9)	24 (20.7)	11 (20.4)	29 (33.3)	0.082
Cyanosis	1 (0.4)	0 (0)	0 (0)	1 (1.1)	-
**Affected lung side**,** n (%)**					
• Bilateral	11 (4.3)	9 (7.8)	1 (1.9)	1 (1.1)	0.015*
• Left	82 (31.9)	44 (37.9)	17 (31.5)	21 (24.1)	
• Right	164 (63.8)	63 (54.3)	36 (66.7)	65 (74.7)	
**Hospital stay (days)**, median (IQR)	10 (6–15)	7 (5–9.75)	16.5 (11–21.25)	13 (8–15)	< 0.001*

*Statistically significant at *p* < 0.05, CAP: community-acquired pneumonia, NP: necrotizing pneumonia, PPE: parapneumonic effusion, RR: respiratory rate, IQR: interquartile range

Among the 87 children with parapneumonic effusion, simple pleural effusion was identified in 38 (43.7%), complex non-septated effusion in 35 (40.2%), and complex septated effusion in 6 (6.9%) on admission. Of these, 58 (66.7%) required pleural intervention. Chest tube drainage alone was the most common intervention (40/58, 69.0%), followed by chest tube drainage with decortication (6/58, 10.3%), decortication alone (4/58, 6.9%), and chest tube drainage with intrapleural urokinase (1/58, 1.7%). Decortication, when performed, was carried out via video-assisted thoracoscopic surgery (VATS) or open thoracotomy, according to the operating surgeon’s judgment and case-specific factors. The remaining patients were managed conservatively.

### Laboratory findings

Baseline laboratory parameters of the studied population are summarized in Table 2 . Mean hemoglobin was 10.03 ± 1.43 g/dL across the cohort and did not differ significantly between groups (*p* = 0.139). The leukocyte differential highlighted a divergence in inflammatory phenotype between groups: absolute neutrophil count (ANC) was highest in PPE (*p* < 0.001), while absolute lymphocyte count (ALC) was paradoxically elevated in NP (*p* = 0.012), a pattern that may reflect the dysregulated or prolonged immune response associated with tissue necrosis.

**Table 4:** Lung ultrasound findings and scores on the first and fifth days among CAP, NP, and PPE groups

Variable	Total (*n* = 257)	CAP (*n* = 116)	NP (*n* = 54)	PPE (*n* = 87)	*P* value
Hemoglobin (g/dL), mean ± SD	10.03 ± 1.43	10.12 ± 1.51	9.64 ± 1.14	10.06 ± 1.64	0.139
TLC (×10³/µL), median (IQR)	18.1 (11.9–24.6)	16.3 (11.2–21.6)	16.8 (10.7–24.8)	20.2 (13.8–28.3)	0.008*
Platelets (×10³/µL), median (IQR)	367 (287–508.5)	326 (259–436)	401.5 (310.3–538.3)	422 (308–562)	< 0.001*
ANC (×10³/µL), median (IQR)	13.3 (8.1–18.2)	12.1 (7.98–17.7)	11.8 (7.36–16.57)	15.1 (9.54–21.25)	< 0.001*
ALC (×10³/µL), median (IQR)	2.86 (1.8–4.38)	2.39 (1.31–3.36)	3.95 (2.4–6.75)	3.15 (2.09–4.76)	0.012*
CRP (mg/L), median (IQR)	96 (49.4–188.3)	96 (48–196)	96 (46.1–162.3)	96 (90–190)	0.181
D-dimer (µg/mL), median (IQR)	1.8 (0.8–13)	1.09 (0.5–2.24)	2.95 (1.28–5)	2.5 (1.57–4.2)	< 0.001*

*Statistically significant at *p* < 0.05, TLC: Total Leukocyte Count, IQR: Interquartile Range, ANC: Absolute Neutrophil Count, ALC: Absolute Lymphocyte Count, NLR: Neutrophil-to-Lymphocyte Ratio, dNLR: Derived Neutrophil-to-Lymphocyte Ratio, SII: Systemic Immune-Inflammation Index, CRP: C-Reactive Protein

Serum CRP was markedly elevated across all groups without significant intergroup difference (*p* = 0.181), underscoring its limited utility for phenotype discrimination. In contrast, D-dimer levels differed significantly (*p* < 0.001), with substantially higher values in NP and PPE than CAP, supporting a role for coagulation activation in complicated pneumonia and providing a laboratory correlate of the higher LUS scores observed in these groups.

### Lung ultrasound findings

Longitudinal LUS assessment between Day 1 and Day 5 demonstrated significant radiological improvement across the cohort (*p* < 0.001). The proportion of patients with isolated B-lines, a marker of partial re-aeration, increased from 35.0% to 54.9% (*p* < 0.001), while consolidation features including the shred sign, air bronchogram, tissue-like pattern, and lung collapse showed significant reductions (all *p* ≤ 0.013) (Supplementary Table 1). Both median LUS score and number of involved lung segments decreased significantly over this interval (both *p* < 0.001). These changes indicate that five days of treatment produces measurable, sonographically detectable lung re-aeration, validating Day 5 LUS as a meaningful monitoring time point (Table 3 ).

At admission, the LUS score and number of involved lung segments were significantly higher in children with NP and PPE compared with CAP (both *p* < 0.001), demonstrating a clear sonographic severity gradient between uncomplicated and complicated pneumonia. These differences persisted at Day 5, with significantly higher LUS scores and segment involvement in NP and PPE compared with CAP (*p* < 0.001), indicating sustained discriminatory ability of LUS during follow-up (Table 4 ).

Qualitative LUS features further supported differentiation of complicated pneumonia phenotypes. At baseline, Tissue-like pattern, heterogeneous hypoechoic areas, lung collapse, hypoechoic lesions, absent lung sliding, and all forms of pleural effusion were significantly more frequent in NP and PPE than in CAP (all *p* < 0.001). NP showed the highest prevalence of parenchymal complications, particularly heterogeneous hypoechoic areas, hypoechoic lesions, and absent lung sliding, reflecting the destructive nature of necrotizing disease at the sonographic level (Supplementary Table 2).

At Day 5, qualitative differences between groups remained evident. CAP patients showed a predominance of isolated B-lines by this point, whereas persistent consolidation signs, shred sign, air bronchogram, tissue-like pattern, and complicated pleural effusions remained significantly more common in NP and PPE (all *p* < 0.001). Heterogeneous hypoechoic areas and absent lung sliding continued to be markedly more prevalent in NP (Supplementary Table 2).

### Diagnostic performance of LUS score

**Fig. 2:**
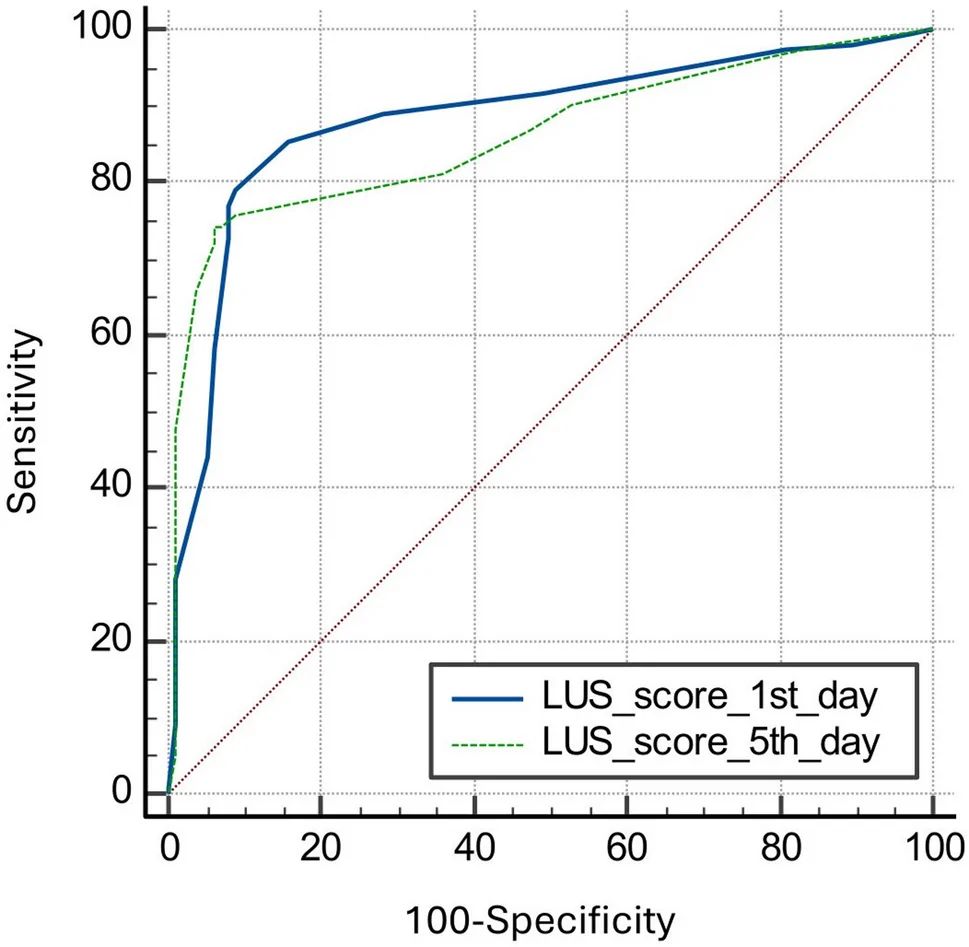
ROC curve showing performance of LUS on 1st and 5th day in prediction of incidence of complications

Internal bootstrap validation demonstrated stable diagnostic performance of the LUS score. For the first-day assessment, the AUC was 0.881 (95% CI: 0.835–0.918), with a bootstrap 95% CI of 0.834–0.923. The optimal cutoff identified by the Youden index was > 8, yielding a sensitivity of 79.02% and a specificity of 91.23%. Similarly, for the fifth-day assessment, the AUC was 0.861 (95% CI: 0.812–0.901), with a bootstrap 95% CI of 0.809–0.903. The same cutoff (> 8) yielded a sensitivity of 74.13% and a specificity of 93.86% (Fig. 2; Table 5).

**Table 5:** Diagnostic performance of lung ultrasound score on the first and fifth days

Time of assessment	Cutoff value	AUC	95% CI	Bootstrap 95% CI	Sensitivity	Specificity	PPV	NPV	*P* value
First day	> 8	0.881	0.835–0.918	0.834–0.923	79.02%	91.23%	91.9%	77.6%	< 0.001*
Fifth day	> 8	0.861	0.812–0.901	0.809–0.903	74.13%	93.86%	93.8%	74.3%	< 0.001*

*Statistically significant at *p* < 0.05, AUC, area under the receiver operating characteristic curve; CI, confidence interval; PPV, positive predictive value; NPV, negative predictive value

### Predictors of LUS score

On multivariable linear regression analysis, LUS score on the first day was independently associated with duration of hospitalization (β = 0.221, *p* < 0.001), D-dimer level (β = 0.334, *p* < 0.001), and absolute neutrophil count (ANC) (β = 0.060, *p* = 0.018).

Similarly, on the fifth day, LUS score remained independently associated with duration of hospitalization (β = 0.215, *p* < 0.001), D-dimer (β = 0.386, *p* < 0.001), and ANC (β = 0.066, *p* = 0.023) (Table 6). The strengthening of the D-dimer association between Day 1 and Day 5 (β 0.334 to 0.386) suggests that persistent sonographic disease at follow-up is increasingly linked to ongoing coagulation activation, a mechanism consistent with the microvascular thrombosis implicated in necrotizing lung injury.

**Table 6:** Multivariable linear regression analysis of factors associated with lung ultrasound score on the first and fifth day

Model	Variable	Unstandardized β	Std. Error	t	95% CI	*P* value
First day	Duration of hospitalization (days)	0.221	0.032	6.849	0.157–0.284	< 0.001*
	D-dimer	0.334	0.081	4.109	0.174–0.494	< 0.001*
	ANC	0.060	0.025	2.373	0.010–0.110	0.018*
Fifth day	Duration of hospitalization (days)	0.215	0.037	5.854	0.143–0.288	< 0.001*
	D-dimer	0.386	0.093	4.168	0.204–0.569	< 0.001*
	ANC	0.066	0.029	2.291	0.009–0.123	0.023*

*Statistically significant at *p* < 0.05, β represents regression coefficients. CI indicates confidence interval. Variables with *p* < 0.05 were considered statistically significant, ANC: Absolute Neutrophil Count

Interobserver agreement for LUS interpretation was substantial to almost perfect across all parameters and time points. On Day 1, Cohen’s κ ranged from 0.72 to 0.89, with the highest agreement observed for absent lung sliding (κ = 0.89) and heterogeneous hypoechoic areas (κ = 0.85); the LUS score itself showed κ = 0.82. Day 5 values were comparable (κ range 0.75–0.91; LUS score κ = 0.84), all *p* < 0.001. The overall κ for discrimination of complicated phenotypes (NP or PPE vs. CAP) was 0.83 (95% CI: 0.76–0.90), indicating that the clinically most consequential LUS judgment, distinguishing complicated from uncomplicated disease, is highly reproducible between trained operators and supports the generalizability of the proposed LUS score threshold.

To assess the impact of disease prevalence on the predictive performance of LUS, PPV and NPV were recalculated across hypothetical prevalence scenarios of complicated pneumonia (5%, 10%, and 20%) using the observed sensitivity and specificity values (Supplementary Table S3). At the first-day assessment, the estimated PPV decreased from 91.9% at the cohort prevalence to 32.2% at a 5% prevalence scenario, while NPV increased from 77.6% to 98.8%. Similarly, at the fifth-day assessment, PPV decreased from 93.8% to 38.9%, whereas NPV increased from 74.3% to 98.6%.

## Discussion

This prospective comparative study provides, to our knowledge, the first simultaneous longitudinal evaluation of quantitative LUS performance across CT-confirmed NP, PPE, and uncomplicated CAP in a single pediatric cohort. Our findings support the potential role of quantitative LUS as a radiation-free bedside tool for differentiating complicated pneumonia phenotypes and monitoring disease evolution during hospitalization.

The clinical and demographic profile of NP patients, younger age, lower weight-for-age z-score, higher respiratory rate, and more frequent grunting, aligns with evidence that necrotizing disease disproportionately affects younger, nutritionally vulnerable children [18–20], though overlapping features between groups underscore the need for objective imaging alongside clinical assessment.

The laboratory profile further demonstrated the difficulty of distinguishing pneumonia phenotypes using inflammatory markers alone. CRP showed limited discriminatory value [21, 22] whereas the association of D-dimer and ANC with LUS score supports the concept that sonographic abnormalities may reflect both local lung injury and systemic inflammatory responses [23–25]. Specific sonographic features associated with complicated disease, including heterogeneous hypoechoic areas, absent lung sliding, and tissue-like consolidation, may reflect parenchymal destruction and pleural involvement and are consistent with previous pediatric LUS studies of complicated pneumonia [26–30]. The quantitative LUS score added discriminatory value beyond qualitative pattern recognition: a score > 8 at admission correctly identified complicated disease in 79.02% of cases, a sensitivity that compares favorably with CXR performance in detecting NP (reported as 60–70% in prior series) and is achieved without radiation or sedation [4, 5].

The high interobserver agreement observed in this study (κ > 0.80) supports the reproducibility of LUS when performed using standardized protocols by trained operators. However, both operators were experienced pediatric pulmonologists with dedicated LUS training and > 500 prior examinations. Comparable performance among less experienced users may require structured training, supervised practice, and competency assessment, which should be evaluated in multicenter studies. A complete 12-zone LUS examination can generally be performed within a few minutes at the bedside, allowing repeated radiation-free assessments during hospitalization. Nevertheless, LUS should complement rather than replace CT when detailed anatomical assessment is required, including confirmation of necrosis or cavitation, evaluation of complex complications, unexplained clinical deterioration, or planning of interventional procedures.

The longitudinal findings of this study address an important gap in the literature regarding the role of LUS in monitoring treatment response in complicated pediatric pneumonia. The observed evolution of sonographic patterns during hospitalization, characterized by improved aeration and reduced consolidation features, is consistent with the healing trajectory described by Elabbas et al. and Ginsburg et al. in pediatric pneumonia cohorts [7, 31]. These findings support the potential value of serial LUS as a bedside tool for assessing disease progression while avoiding repeated radiation exposure.

The reduction in LUS score and involved lung segments over time reflects its ability to quantify disease evolution, with the score’s diagnostic role shifting from identifying complicated disease at admission to confirming persistent disease at follow-up.

In practical terms, a persistent LUS score > 8 on Day 5 was associated with high specificity (> 93%) for complicated disease as defined at admission. However, because contemporaneous CT was not available for all patients at Day 5, this finding should be interpreted cautiously and requires validation against an appropriate follow-up reference standard before being used to guide management decisions.

Previous studies have demonstrated the utility of quantitative LUS scoring for assessing disease severity and reducing radiation exposure in pediatric respiratory disorders [32, 33]; however, evidence specifically addressing pediatric community-acquired pneumonia remains limited [34]. The present study extends these observations by evaluating a standardized quantitative LUS approach across distinct pneumonia phenotypes and during follow-up. Although these findings support the potential clinical utility of LUS, external validation is required before widespread adoption of a specific cutoff.

The association between LUS score and clinical outcomes supports its potential role as more than an imaging marker. The relationship between higher LUS scores, prolonged hospitalization, inflammatory markers, and coagulation activation suggests that LUS may reflect the overall severity of pulmonary and systemic disease processes [10, 15, 35–37].

The prevalence of complicated pneumonia in the present cohort was relatively high because the study was conducted in a tertiary referral setting enriched with severe and complicated cases. This may affect the predictive values of LUS, as PPV and NPV depend on disease prevalence. Our prevalence-based analysis demonstrated that the clinical interpretation of positive and negative results may vary substantially across different settings. Therefore, the proposed cutoff should be applied cautiously in populations with different disease prevalence, and validation in primary and secondary care settings is warranted.

Several limitations of this study merit consideration. First, the single-center design at a tertiary referral unit with a high prevalence of complicated pneumonia may limit generalizability to populations with different pneumonia epidemiology, vaccination coverage, and microbiological profiles. Second, chest CT was performed only when clinically indicated and was not available for all children with PPE. Therefore, an underlying NP component could not be completely excluded in children without CT, potentially resulting in misclassification between the PPE and NP categories. This should be considered when interpreting diagnostic and comparative analyses. In addition, CT was not routinely repeated at Day 5 because of radiation concerns; consequently, no contemporaneous imaging reference standard was available for the Day 5 assessment, introducing potential verification bias. The Day 5 findings therefore require validation against an appropriate follow-up reference standard. Third, the five-day follow-up captures early in-hospital response but does not address longer-term sonographic evolution, post-discharge monitoring, or outcomes beyond hospitalization. Fourth, individual pneumococcal vaccination status was not systematically collected and therefore could not be evaluated. Fifth, no formal a priori sample size calculation was performed. Although consecutive enrollment resulted in a relatively large prospective cohort (*n* = 257), unequal group sizes may have limited statistical power for some subgroup comparisons.

Finally, while internal bootstrap validation demonstrated a stable cutoff (> 8) with minimal optimism in AUC estimation, external prospective validation in an independent cohort remains necessary before this threshold can be generalized to other populations or clinical settings.

## Conclusion

In children hospitalized with CAP, a quantitative LUS score threshold of > 8 shows promising diagnostic performance for identifying complicated pneumonia, including necrotizing pneumonia and parapneumonic effusion, at admission and for detecting persistent complicated disease at Day 5 follow-up with high specificity (93.86%). These findings suggest that LUS may serve as a valuable radiation-free bedside tool for identifying and monitoring complicated pneumonia. However, LUS should not be considered a substitute for CT when further characterization of complicated disease is clinically required, particularly to differentiate PPE from necrotizing pneumonia or lung abscess. The consistent independent association of the LUS score with hospitalization duration and D-dimer at both time points further supports its potential prognostic value. Although internal bootstrap validation demonstrated the stability of the proposed cutoff, prospective multicenter studies with external validation are required before its routine clinical implementation.

## Supplementary Information

Below is the link to the electronic supplementary material.

Supplementary Material 1

## Data Availability

The datasets used and/or analyzed during the current study are available upon reasonable request from the corresponding author.

## Abbreviations

AUC: Area under the curve
ANC: Absolute neutrophil count
ALC: Absolute lymphocyte count
BCa: Bias-corrected and accelerated
CAP: Community-acquired pneumonia
CI: Confidence interval
CRP: C-reactive protein
CT: Computed tomography
CXR: Chest radiograph
IQR: Interquartile range
LUS: Lung ultrasound
NP: Necrotizing pneumonia
NPV: Negative predictive value
PPE: Parapneumonic effusion
PPV: Positive predictive value
ROC: Receiver operating characteristics
SD: Standard deviation
