# Supplementary material for: Clinical utility of lung ultrasound in differentiating and monitoring complicated pediatric pneumonia: a prospective diagnostic accuracy study
Source: Ital J Pediatr. 2026 Oct 3;52:147. doi: 10.1186/s13052-026-02341-3 (PMC13640073; doi:10.1186/s13052-026-02341-3)
Supplement: Supplementary file 1 [file 13052_2026_2341_MOESM1_ESM.pdf]

Supplementary Table 1. Comparison of lung ultrasound findings and scores between the first and fifth days of admission

Lung ultrasound parameter	First day	Fifth day	*p* value	BH-adjusted q value
**Qualitative findings, n (%)**				
Separate B-lines	90 (35.0)	141 (54.9)	<0.001*	<0.001*
Confluent B-lines	196 (76.3)	191 (74.3)	0.653	0.762
Shred sign	228 (88.7)	144 (56.0)	<0.001*	<0.001*
Air bronchogram	236 (91.8)	185 (72.0)	<0.001*	<0.001*
Tissue-like pattern	105 (40.9)	30 (11.7)	<0.001*	<0.001*
Heterogenous hypoechoic areas (HHA)	24 (9.3)	50 (19.5)	<0.001*	<0.001*
Lung collapse	20 (7.8)	9 (3.5)	0.013*	0.020*
Hypoechoic lesions	11 (4.3)	12 (4.7)	0.999	1.000
Absent lung sliding	14 (5.4)	15 (5.8)	0.999	0.999
Simple pleural effusion	50 (23.3)	46 (17.9)	0.018*	0.025*
Complex non-septated effusion	52 (20.2)	46 (17.9)	0.238	0.303
Complex septated effusion	14 (5.4)	25 (9.7)	0.003*	0.005*

*Statistically significant at raw $p < 0.05$ and remained significant after Benjamini-Hochberg false discovery rate correction (Q = 0.05). IQR: interquartile range.

Supplementary Table S2. Qualitative lung ultrasound findings on the first and fifth days among CAP, NP, and PPE groups

Lung ultrasound parameter	CAP (n = 116)	NP (n = 54)	PPE (n = 87)	*P* value	BH-adjusted q value
**First day findings, n (%)**					
Separate B-lines	48 (41.4)	17 (31.5)	25 (28.7)	0.145	0.150
Confluent B-lines	95 (81.8)	37 (68.5)	64 (73.6)	0.124	0.134
Shred sign	98 (84.5)	48 (88.9)	82 (94.3)	0.093	0.109
Air bronchogram	105 (90.5)	49 (90.7)	82 (94.3)	0.597	0.597
Tissue-like pattern	25 (21.6)	35 (64.8)	45 (51.7)	<0.001*	<0.001*
Heterogenous hypoechoic areas (HHA)	0 (0)	17 (31.5)	7 (8.0)	<0.001*	<0.001*
Lung collapse	3 (2.6)	5 (9.3)	12 (13.8)	0.012*	0.015*
Hypoechoic lesions	0 (0)	8 (14.8)	3 (3.4)	<0.001*	<0.001*
Absent lung sliding	0 (0)	11 (20.4)	3 (3.4)	<0.001*	<0.001*
Simple pleural effusion	10 (8.6)	12 (22.2)	38 (43.7)	<0.001*	<0.001*
Complex non-septated effusion	1 (0.9)	16 (29.6)	35 (40.2)	<0.001*	<0.001*
Complex septated effusion	1 (0.9)	7 (13.0)	6 (6.9)	0.004*	0.005*
**Fifth day findings, n (%)**					
Separate B-lines	82 (70.7)	17 (31.5)	42 (48.3)	<0.001*	<0.001*
Confluent B-lines	89 (76.7)	32 (59.3)	70 (80.5)	0.014*	0.017*
Shred sign	41 (35.3)	39 (72.2)	64 (73.6)	<0.001*	<0.001*
Air bronchogram	62 (53.4)	44 (81.5)	79 (90.8)	<0.001*	<0.001*
Tissue-like pattern	3 (2.6)	14 (25.9)	13 (14.9)	<0.001*	<0.001*
Heterogenous hypoechoic areas (HHA)	3 (2.6)	39 (72.2)	8 (9.4)	<0.001*	<0.001*
Lung collapse	2 (1.7)	1 (1.9)	6 (6.9)	0.106	0.119
Hypoechoic lesions	1 (0.9)	8 (14.8)	3 (3.4)	<0.001*	<0.001*
Absent lung sliding	2 (1.7)	11 (20.4)	2 (2.3)	<0.001*	<0.001*
Simple pleural effusion	7 (6.0)	10 (18.5)	29 (33.3)	<0.001*	<0.001*
Complex non-septated effusion	1 (0.9)	17 (31.5)	28 (32.2)	<0.001*	<0.001*
Complex septated effusion	1 (0.9)	9 (16.7)	15 (17.2)	<0.001*	<0.001*

*Statistically significant at raw p < 0.05 and remained significant after Benjamini-Hochberg false discovery rate correction (Q = 0.05). IQR: interquartile range.

Supplementary Table S3 Estimated Positive and Negative Predictive Values of the LUS Cutoff at Different Prevalence Scenarios of Complicated Pneumonia

Time of assessment	Assumed prevalence of complicated pneumonia	PPV (%)	NPV (%)
**First day (Sensitivity 79.02%, Specificity 91.23%)**	Cohort prevalence	91.9	77.6
	5%	32.2	98.8
	10%	50.0	97.5
	20%	69.3	94.6
**Fifth day (Sensitivity 74.13%, Specificity 93.86%)**	Cohort prevalence	93.8	74.3
	5%	38.9	98.6
	10%	57.3	97.0
	20%	75.1	93.6

PPV, positive predictive value; NPV, negative predictive value, Positive predictive value (PPV) = (Sensitivity × Prevalence) / [(Sensitivity × Prevalence) + ((1 − Specificity) × (1 − Prevalence))], Negative predictive value (NPV) = [Specificity × (1 − Prevalence)] / [(Specificity × (1 − Prevalence)) + ((1 − Sensitivity) × Prevalence)].
